# An Interpretable Three-Dimensional Artificial Intelligence Model for Computer-Aided Diagnosis of Lung Nodules in Computed Tomography Images

**DOI:** 10.3390/cancers15184655

**Published:** 2023-09-21

**Authors:** Sheng-Chieh Hung, Yao-Tung Wang, Ming-Hseng Tseng

**Affiliations:** 1 Master Program in Medical Informatics, Chung Shan Medical University, Taichung 402, Taiwan; s0859003@gm.csmu.edu.tw; 2 School of Medicine, Chung Shan Medical University, Taichung 402, Taiwan; cshy1025@gmail.com; 3 Division of Pulmonary Medicine, Department of Internal Medicine, Chung Shan Medical University Hospital, Taichung 402, Taiwan; 4 Department of Medical Informatics, Chung Shan Medical University, Taichung 402, Taiwan; 5 Information Technology Office, Chung Shan Medical University Hospital, Taichung 402, Taiwan

**Keywords:** artificial intelligence, lung cancer, CT images, neural network, deep learning, interpretable

## Abstract

**Simple Summary:**

This study presents an AI system for the automatic diagnosis of lung cancer based on lung nodule images from CT scans. Lung cancer is the leading cause of cancer-related deaths in Taiwan, and early detection can improve the survival rate of patients. The system uses a 3D interpretable hierarchical semantic convolutional neural network (HSNet) that can recognize different features of lung nodules, such as calcification, margin, texture, sphericity, and malignancy. The system achieves better performance than previous methods, with high accuracy and interpretability.

**Abstract:**

Lung cancer is typically classified into small-cell carcinoma and non-small-cell carcinoma. Non-small-cell carcinoma accounts for approximately 85% of all lung cancers. Low-dose chest computed tomography (CT) can quickly and non-invasively diagnose lung cancer. In the era of deep learning, an artificial intelligence (AI) computer-aided diagnosis system can be developed for the automatic recognition of CT images of patients, creating a new form of intelligent medical service. For many years, lung cancer has been the leading cause of cancer-related deaths in Taiwan, with smoking and air pollution increasing the likelihood of developing the disease. The incidence of lung adenocarcinoma in never-smoking women has also increased significantly in recent years, resulting in an important public health problem. Early detection of lung cancer and prompt treatment can help reduce the mortality rate of patients with lung cancer. In this study, an improved 3D interpretable hierarchical semantic convolutional neural network named HSNet was developed and validated for the automatic diagnosis of lung cancer based on a collection of lung nodule images. The interpretable AI model proposed in this study, with different training strategies and adjustment of model parameters, such as cyclic learning rate and random weight averaging, demonstrated better diagnostic performance than the previous literature, with results of a four-fold cross-validation procedure showing calcification: 0.9873 ± 0.006, margin: 0.9207 ± 0.009, subtlety: 0.9026 ± 0.014, texture: 0.9685 ± 0.006, sphericity: 0.8652 ± 0.021, and malignancy: 0.9685 ± 0.006.

## 1. Introduction

Lung cancer is a malignant tumor that grows in the bronchus or alveoli. There are no obvious symptoms in the early stages, and traditional lung X-rays are generally used for rapid clinical examination. However, due to their low sensitivity and difficulty in detecting tumors smaller than 1 cm in diameter, low-dose computed tomography (LDCT) is currently considered a more sensitive tool for early detection. According to the National Institutes of Health, LDCT can reduce lung cancer mortality by 20% compared to traditional X-ray examinations [1]. If non-small-cell lung cancer can be surgically removed in the first stage, the 5-year survival rate can reach between 80% and 90%. On the contrary, if it is detected only at the late stage, the 5-year survival rate drops to only about 10%, and the prognosis worsens with each stage. Therefore, if there is a method for early detection of lung cancer and prompt surgical treatment after the lesion is discovered, it can significantly reduce the mortality rate of patients with lung cancer.

According to the 2020 version of the GLOBOCAN research, 60% of newly diagnosed lung cancer was found in Asia, and 62% of all deaths due to lung cancer were also in Asia [2]. Lung cancer in the non-smoking population is increasing worldwide, especially in East Asia [2]. In Taiwan, the standardized death rate of lung cancer in 2022 was 21.8 per 100,000 population, ranking it as the leading cause of cancer-related mortality in Taiwan. It accounts for approximately one-fifth of all cancer deaths, with more than half of mortality patients not having exposure to smoking [2,3]. Early detection of lung cancer is the most effective mode to improve cancer survival, and LDCT has confirmed the efficacy of lung cancer screening. Starting in July 2022, Taiwan has provided subsidized LDCT screenings every two years for people at high risk for lung cancer.

Currently, doctors use LDCT scans to detect lung cancer. Identification of lung cancer depends largely on the experience of the physician, and inexperienced physicians can make diagnostic errors or overlook important details. Deep learning models can automatically process images, and their application to clinical diagnosis and treatment in medical imaging is rapidly developing. With computer-assisted diagnosis, less experienced physicians can perform an initial screening.

In lung-cancer-related examinations, using AI models to detect nodules in lung CT images is a promising method to assist doctors in diagnosis. To alleviate the burden of manual loading by physicians with a large number of LDCT screenings, the development of an AI model with interpretable capabilities for computer-aided diagnosis of lung nodules becomes a crucial research topic [3,4,5,6].

Although deep learning models have the ability to produce impressive classification outcomes, their ‘black-box’ nature makes it challenging to comprehend the derivation of these results. Consequently, interpreting the reasoning mechanisms of deep learning models has emerged as a significant area of research. Current XAI (explainable artificial intelligence) models can be broadly categorized into three types based on their explainability techniques [7]: (1) pre-modeling explainability, which focuses primarily on the analysis of sample trends; (2) explainable modeling, which involves the development of inherently interpretable models; and (3) post-modeling explainability, which mainly pertains to methods for post hoc inference of model prediction results. Pulmonary nodule images may exhibit some semantic features such as sphericity, margin, subtlety, texture, and calcification. These features provide useful information for physicians to diagnose whether the pulmonary nodules are benign or malignant [8]. Therefore, the ‘Interpretable AI Model’ that this study aims to develop falls under explainable modeling. Users only need to input the images of the pulmonary nodule, and the model can simultaneously output these five semantic features of the images and the predicted category of benign or malignant.

## 2. Related Work

Computer systems have been applied to medical care as an aid to physicians for a long time. Computer-aided diagnosis (CADx) can help physicians understand the information in the images to assist in analysis and evaluation. In 1998, Kanazawa proposed a computer-aided automatic diagnosis system for lung cancer detection [9]. CAD systems can analyze various sections extracted from the images and find regions of interest. However, the feature extraction methods required for CAD systems were mostly manually processed by radiologists at that time. Although it could detect lesions more easily than a single inspection method, CAD systems were limited by the time-consuming data preprocessing and computational limitations of computer performance, making it difficult to use as a routine application in clinical practice.

As computer performance improved and optimized algorithms were proposed, computer-aided diagnosis gradually became a popular topic in medical imaging diagnosis research [10,11]. The important purpose of creating a CAD system for classification is to effectively capture the regions of interest in the image and process the features [12]. Typically, the system is divided into two main tasks. The first task is to detect regions of interest, which will detect all the required feature blocks from the CT scan images. If all the feature blocks are recognized, there may be a large number of false positives, so the second task is to classify the previously detected candidate feature regions as benign or malignant. This can reduce the large number of false-positive areas generated in the first task and can further analyze the classified data in detail. In 2015, Devinder Kumar et al. attempted to establish a CAD system consisting of an autoencoder and a binary decision tree for the benign and malignant classification of lung nodules using the entire lung CT slice, with an overall accuracy of 75% [13].

The development of computer-aided lung nodule diagnosis can be roughly divided into three stages [5]. The initial stage was largely based on traditional image processing techniques. Later, machine learning methods were introduced to use a small amount of data to build models. Recently, due to the fact that deep learning can optimize the training of convolutional neural networks by collecting a large amount of data, the accuracy of the model has been effectively improved [14,15,16,17,18,19]. Numerous research works have demonstrated the many advantages of CNN in lung nodule detection and lung cancer diagnosis within CT images. First, CNN enables an automated diagnostic and classification process, thus saving valuable time for medical professionals and improving diagnostic efficiency. Second, CNN significantly improves diagnostic accuracy. Third, it reduces the risk of human error in expert judgment.

The research system to apply deep learning to the diagnosis of lung nodules can be divided into three modules [3]: nodule detection, nodule segmentation, and nodule classification. The detection module is responsible for the detection and positioning of nodules, the segmentation module is responsible for drawing the contours of the nodule voxels, and the classification module mainly predicts whether the nodule belongs to benign or malignant types. In recent years, many studies have been devoted to applications related to deep learning. Recently, there have been four review articles on computer-aided lung nodule diagnosis that can provide readers with further references [3,4,5,6].

Training on the entire lung slice may cause confusion in image judgment due to the loss or blurring of feature details caused by compressed image data or interference from a large area of the image with too much noise. This may increase the difficulty of computer vision in lung cancer diagnosis, and misjudgments may further waste the medical resources required for re-testing. Convolutional neural network models can filter out the features of interest in the image through feature training and can therefore be applied to image classification. Cutting out fixed-size feature samples from lung CT images and training them using convolutional neural networks has gradually become a mainstream method [20,21,22], and three-dimensional medical images are also possible to efficiently compute and analyze. Joshua et al. used the improvised 3D AlexNet with lightweight architecture for the detection of lung cancer [23].

In 2019, Shen et al. [8] proposed a hierarchical semantic convolutional neural network (HSCNN) to classify nodules in three-dimensional medical images. The HSCNN model consists of three parts: a 3D convolutional feature learning model, a low-level classification task, and a high-level classification task. The model takes in fixed-size three-dimensional nodule images as input and extracts nodule features in the 3D convolutional feature learning model. These features are then fed into the low-level classification task, which predicts five different semantic labels. Finally, the high-level classification task integrates the nodule features and the semantic features of the five classification tasks to predict nodule malignancy. Shen et al. (2019) evaluated the HSCNN model using a four-fold cross-validation procedure and reported the AUC results for each semantic label. The AUC results were as follows: calcification, 0.930 ± 0.034; margin, 0.776 ± 0.033; subtlety, 0.803 ± 0.015; texture, 0.850 ± 0.042; sphericity, 0.568 ± 0.015; and malignancy, 0.856 ± 0.026 [8].

These research findings indicate that using CNN as an assistive tool in lung cancer diagnosis is viable and holds potential clinical value. However, most prior studies have developed black-box AI models. In contrast, this study builds upon the transparent HSCNN white-box model [8] for further improvement and validation, ensuring its reliability and accuracy in clinical applications.

## 3. Materials and Methods

### 3.1. Lung Image Database Consortium Image Collection

The image dataset used in this study is the lung nodule feature image set provided by Shen et al. [8]. The feature images were extracted from the Lung Image Database Consortium image collection (LIDC-IDRI) [24], a freely available image dataset collected in collaboration with eight medical imaging companies and seven academic research centers, and archived in the Cancer Image Archive (TCIA).

**Figure 1:**
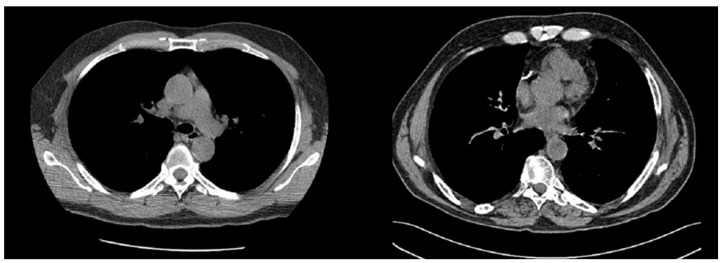
CT slice DICOM images [24].

The LIDC-IDRI dataset contains clinical chest CT scan images and related extensible markup language (XML) files with annotations from four chest radiologists. The dataset includes a total of 1018 case records involving 1010 different patients, including 8 patients who underwent different scans for the second time. The scan images are stored in DICOM format, and the total size of the images is 124 GB. Figure 1 shows CT slice images from the LIDC-IDRI dataset, and we present two different DICOM medical images of CT slices from different patients [24]. In the left image, the slice is positioned at −69.36 mm depth, with a window level of 40 and a window width of 400. In the right image, the slice is positioned at −182.40 mm depth, with a window level of 30 and a window width of 350.

### 3.2. Our Usage of the LIDC-IDRI Dataset

In this paper, we refer to the approach used by Shen et al. [8] and re-categorize the six different semantic feature labels mentioned above into two categories, making it easier to conduct preliminary diagnostic screening using classification prediction labels. The malignancy category is changed from the original five levels to a negative level ranging from “Highly unlikely” to “Indeterminate”, and any level above “Moderately suspicious” is deemed positive. The margin category is categorized as negative for the first to third levels and positive for the fourth and fifth levels of the original data. Similarly, the sphericity and texture categories are categorized as negative for the first to third levels and positive for the fourth and fifth levels of the original data. The calcification category is considered positive only for the “Absent” category, while all other levels are considered negative. The categorized labels used in this study are shown in Table 1.

### 3.3. Interpretable Hierarchical Semantic Convolutional Neural Networks

**Figure 2:**
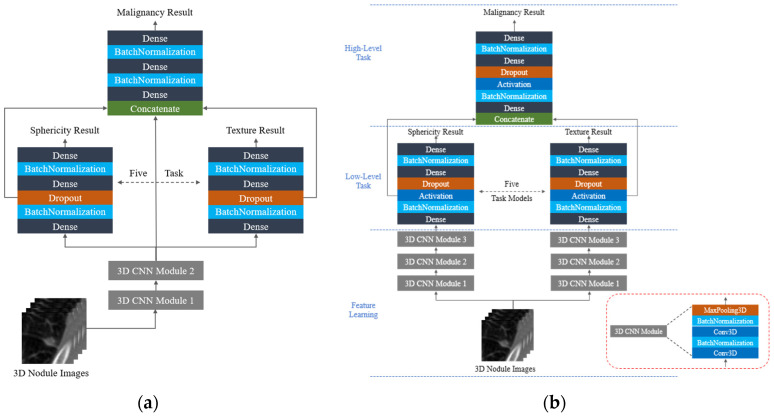
Interpretable hierarchical semantic convolutional neural networks: (**a**) HSCNN [8], (**b**) HSNet.

Based on the network architecture referenced by Shen et al. [8], this study proposes an improved interpretable network architecture for performance testing, as shown in Figure 2b. Figure 2a shows the HSCNN model [8], which uses only five semantic-level classification features after performing a semantic-level classification task to enter a disease-level classification task. Furthermore, this study believes that different semantic classification training should train the features needed for each individual training. Therefore, we attempted to modify the original shared three-dimensional convolution feature extraction model into five individual three-dimensional convolution feature extraction models, each connected to a semantic-level classification task, to investigate whether using different feature extraction models can increase the predictive ability of the model. We also attempted to deepen the network architecture and use a deeper network architecture to obtain more subtle features for semantic-level classification tasks. Furthermore, we changed the extraction of features from the dropout layer to extracting features from the activation function layer during training to reduce the impact of discarded neural element parameters on feature extraction. This model is called HSNet, and the structure of the model is shown in Figure 2b.

### 3.4. Experimental Setup and Evaluation Measures

In this study, we used the TensorFlow 2.7 suite in Python as the platform for our experiment. The hardware and system configuration consisted of a Windows 10 version 22H2 operating system. We employed an NVIDIA RTX 3060 graphics card and an AMD Ryzen 3 PRO 4350G 3.80 GHz 8-core central processing unit (CPU) with 32 GB of memory. The Sklearn package in Python was used to randomly group the data using the train_test_split function, resulting in four sets of data with equal numbers of samples. This study will conduct a 4-fold cross-validation and select the best fold for subsequent diagnostic subtitle training.

The original image set consisted of 4252 images [8]. In this study, we used the 4-fold cross-validation method to randomly split the image set into training and test sets at a ratio of 3:1. Considering that the number of images in the training set is relatively small, overfitting may occur during training. To address this issue, we used data augmentation to generate more training samples from the original training set. Specifically, we randomly transposed and flipped the three axes of the 3D images, increasing the training data by six times to improve the predictive generalization ability of the deep learning model after training. It is worth noting that the test set in this study remained unchanged and was not expanded. In this study, the proposed AI model underwent a training period of 35.32 h, spanning across 300 epochs. The training process was validated using a 4-fold cross-validation method.

And will perform classification verification on the test set and use a confusion matrix to evaluate the classification performance. The confusion matrix can be applied to binary or multi-class classification performance analysis. After the model makes predictions, each category is classified separately and organized into a coherent table to provide a display of data. The classification results of each category can be observed quickly after prediction. The accuracy of the results will be analyzed using the overall accuracy method, which divides the total number of correctly classified images by the total number of test images. This method can be used as a performance analysis method for feature image classification by the model after training. This evaluation method can serve as an effective reference for the results. During the evaluation, the disease-level task classification results of the trained HSCNN model will be used, and the malignancy semantic label for benign and malignant classification will be used as the main indicator for model evaluation.

In addition to comparing the test accuracy, the ROC curve method can also be used as a judging criterion for the screening ability of the classification results to determine the quality of the classification. The larger the area under the receiver operating characteristic curve (AUC), the higher the prediction accuracy. The horizontal axis of the ROC curve chart is the false positive rate (FPR), which represents the ratio of false positive nodules to all benign nodules that are wrongly predicted as malignant. The lower the FPR, the more accurately the model can identify malignant nodules. The vertical axis is the true positive rate (TPR), which represents the ratio of correctly detected malignant nodules to all malignant nodules. The higher the TPR, the more accurate the model’s judgments are. At the same time, the individual AUC label of each test group represents the probability that the classification model can correctly judge positive samples, which is higher than the probability of judging negative samples when randomly selecting a positive sample. The higher the AUC value, the higher the classification accuracy.

Accuracy, AUC, sensitivity, and specificity are widely used in medicine to represent the results of binary classification. Accuracy identifies the correctness of the observation of different class patterns. Sensitivity is the proportion of positive samples that are correctly judged, while specificity represents the proportion of negative samples that are correctly judged. In this paper, accuracy, AUC, sensitivity, and specificity are used as evaluation indicators for semantic-level classification results.

This study compares the predictive performance of two classifiers to determine whether there is a statistically significant difference between them. We use Equation (1) to perform a significant difference test comparison [25], where *E*_1_ and *E*_2_ represent the error rates of Model 1 and Model 2, respectively. The *q* value is represented by (*E*_1_ + *E*_2_)/2 while *n*_1_ and *n*_2_ represent the test set sizes of Model 1 and Model 2, respectively. After a significant difference comparison, if *Ps* ≥ 1.96, it can be considered that the test performance between Model 1 and Model 2 has significant differences with 95% confidence.

(1) $$P_{S}=\frac{\left|{E_{1}-E}_{2}\right|}{\sqrt{q\left(1-q\right)\left(\frac{1}{n_{1}}+\frac{1}{n_{2}}\right)}}$$

## 4. Results

### 4.1. Effect of Optimization Strategies

In our study, we investigate the impact of optimization strategies on the performance of the proposed HSNet model. Specifically, we introduce the early stopping strategy to the training process. This strategy employs a callback method to continuously monitor the accuracy of the main prediction layer on the test set at each iteration. The early stopping strategy halts the training process when it observes no further improvement in accuracy. The primary objective behind this strategy is to prevent overfitting, ensuring that the model does not excessively adapt to the training data.

It is important to note that the choice of when to stop training, based on this accuracy metric, can have varying effects depending on the specific task and loss function used. Therefore, our paper conducts a comprehensive evaluation by comparing test accuracy using models stopped by the early stopping strategy and models trained for a fixed number of iterations (e.g., 300 iterations).

**Figure 3:**
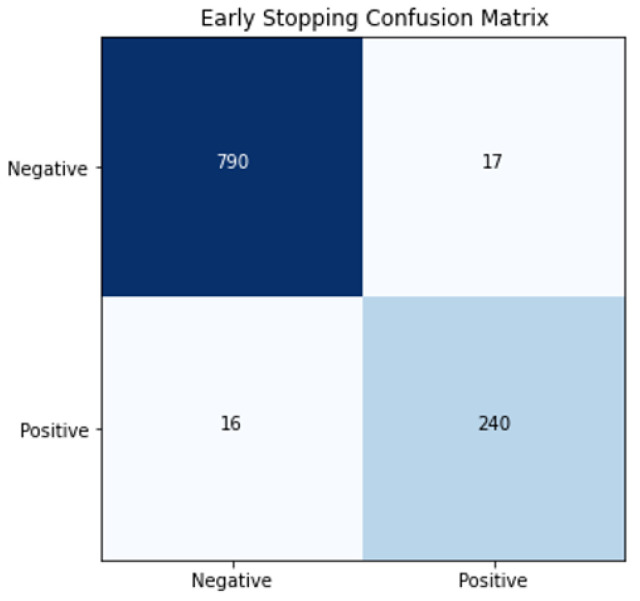
Confusion matrix of HSNet in Fold1 using early stopping.

To implement the early stopping strategy, we employ TensorFlow’s callback method, specifically the ModelCheckpoint callback. This callback is configured to monitor the ‘val_main_prediction_layer_accuracy’ metric and saves model weights whenever an improvement in accuracy is detected. The primary aim of this callback is to ensure that we capture the model’s best performance during training, thus contributing to the prevention of overfitting. The accuracy of the malignancy classification in the prediction of Fold 1 was 96.89%, and the confusion test matrix for Fold 1 generated by the prediction is shown in Figure 3.

**Figure 4:**
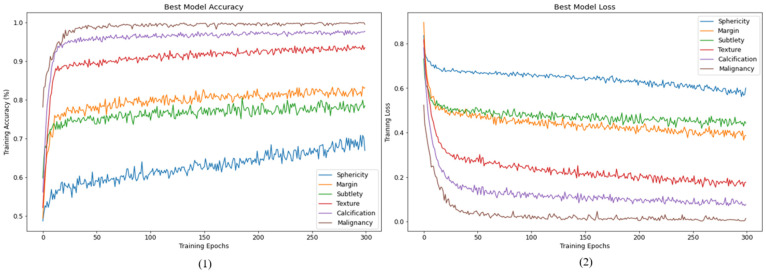
Training history of HSNet: (**1**) accuracy, (**2**) loss.

**Figure 5:**
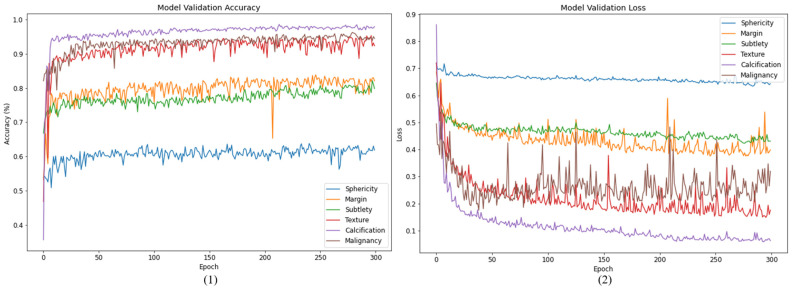
Validation history of HSNet: (**1**) accuracy, (**2**) loss.

The changes in accuracy and loss values during the 300 iterations of this experiment are presented in Figure 4 for the training set. The corresponding results for the validation set are demonstrated in Figure 5.

In addition to adjusting the dropout rate, this study further explores the use of optimization-assisted training other than model parameters. First, we attempted to incorporate the optimization of the cyclical learning rate [26]. The cyclical learning rate (CLR) method sets the learning rate of the model cyclically, varying the training by adjusting the learning rate between the upper bound (Max_lr) and the lower bound (Base_lr) instead of monotonically decreasing the learning rate to achieve the best-fitting state of the model.

**Figure 6:**
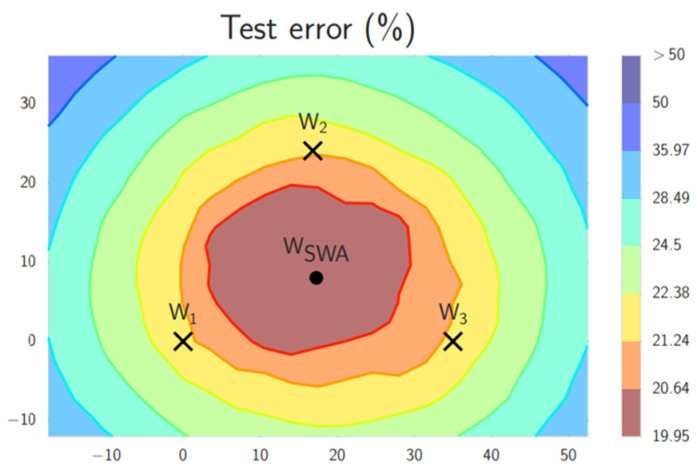
Convergence of SWA weights.

Next, this study attempted to incorporate stochastic weight averaging (SWA), an optimizer package in Tensorflow Addons. SWA [27] can assist the stochastic gradient descent optimizer (SGD) in training. The paper indicated that it could improve the learning generalization ability. The training of SWA uses the cyclical learning rate, allowing the SGD optimizer to explore and converge to the optimal solution. The training process is divided into two stages, during which the model parameters’ weights are averaged at the end of each epoch. Two models are temporarily stored during the training process: the weight of the current epoch training model W_SWA_ and the weight of the previous training model W. The two weights are averaged and used as the model for the next training average, with the number of models represented by n, as shown in Equation (2). It can be understood that weighted averaging is performed with the weights of the model and the previous training to achieve more accurate convergence, helping the model reach the optimal solution more quickly. SWA can average multiple weights (W1, W2, W3) to achieve more accurate convergence, as shown in Figure 6.

(2) $$W_{SWA}=\frac{W_{SWA}\cdot n_{models}+W}{n_{models}+1}$$

To analyze the effect of incorporating optimization methods in training, Run1 uses the SGD optimizer for simple training, Run2 uses the Adam optimizer for training, Run3 adds CLR to adjust the learning rate when using the SGD optimizer for training, Run4 adds CLR to adjust the learning rate when using the SGD optimizer for training while also using the SWA optimization method, and Run5 attempts to use the RMSProp optimizer and CLR to adjust the learning rate. Training results are shown in Table 2.

This paper argues that the use of both the CLR and the SWA optimization methods can result in better prediction accuracy of the model test set. As shown in Table 2, the results of the comparative experiment, compared to the original Adam optimizer, training the model with the SGD optimizer using the CLR method can achieve the best prediction accuracy of the test set, and incorporating the SWA optimization method can further improve the predictive ability of the model.

**Figure 7:**
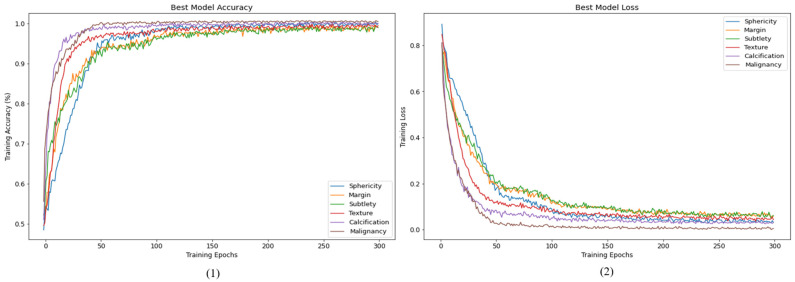
Training history of HSNet with CLR and SWA: (**1**) accuracy, (**2**) loss.

After understanding the potential of the optimization methods mentioned above to improve model training, this paper added CLR and SWA optimization methods and retrained the model using the dataset. The prediction accuracy of the test set (Fold1) can reach 97.83%. The changes in accuracy and loss values during 300 iterations of Fold2 + Fold3 used as the training set in this experiment are shown in Figure 7.

**Figure 8:**
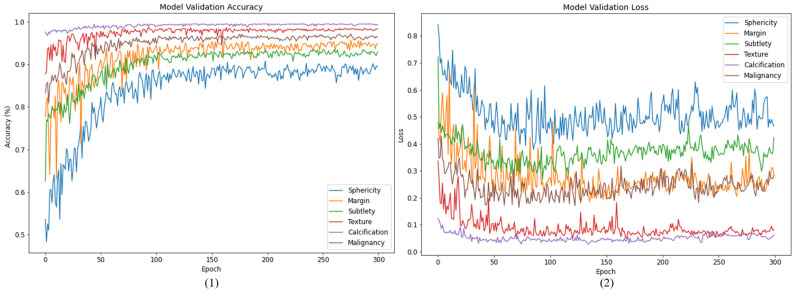
Validation history of HSNet with CLR and SWA: (**1**) accuracy, (**2**) loss.

After using the optimization methods mentioned above, it can be seen in Figure 7 that the training convergence has better accuracy and loss convergence compared to Figure 4. Similarly, when the results of the Fold 4 validation set are displayed in 300 iterations of training, the histories of accuracy and loss values are shown in Figure 8.

### 4.2. Prediction Performance

After obtaining the optimal training method, this study conducted four-fold cross-validation. The deep learning model will use the SGD optimizer from the CLR method and the SWA optimization method during training. First, the best weight training model was obtained by conducting parameter tests of the early stopping strategy using the validation set. Second, the accuracy of the prediction of the classification of the malignancy label was tested using the test set, as shown in Table 3.

In addition to the test accuracy comparison in Table 3, this study can also draw the ROC curve of the true positive rate and false positive rate and judge the quality of the classification results by calculating the AUC value. The corresponding AUC values are shown in Table 4. As can be seen from the comparison results in Table 4, when judging the malignancy of lung nodules, the best results can be achieved using the malignancy semantic label in Fold1 as the test set.

Table 5 shows the average accuracy, sensitivity, and specificity of different semantic labels in four-fold validation, along with their standard deviation (SD). The standard deviation is often used to measure the degree of dispersion of numerical values. The size of the standard deviation can indicate whether there is a difference between most numerical values and their mean value. The results in Table 5 show that the standard deviation of sensitivity and specificity for four-fold validation are both less than 0.05, and there is no significant difference from the mean value.

To assess the significance of model performance improvement, we also trained the HSCNN model using four-fold training and obtained the best model training weights. We evaluated the results of both the HSCNN and HSNet models using paired-sample *t*-tests. The first group of data is the AUC scores of the four-fold validation of the HSNet model, and the second group of data is the AUC scores of the four-fold validation of the HSCNN model. We validated them using six semantic labels, and the results are shown in Table 6, Table 7, Table 8, Table 9, Table 10 and Table 11.

Table 6, Table 7, Table 8, Table 9, Table 10 and Table 11 show the statistical significance of the improvements from the HSNet model compared to the HSCNN model. The null hypothesis is that there is no difference in the average AUC score between the two models. Compared to the results of the HSCNN model in Table 6, Table 7, Table 8, Table 9, Table 10 and Table 11, the HSNet model proposed in this study obtained a significant *p*-value of less than 0.05 on five semantic labels, namely, sphericity, margin, subtlety, texture, and calcification. Therefore, the null hypothesis is rejected, and it can be considered that the HSNet model has a better semantic label prediction ability than the HSCNN model. Consequently, it is believed that when training the malignancy of nodules with the HSNet model, it can better improve the predictive capacity of other semantic features. This finding is of great significance for aiding in the medical diagnosis.

Table 12 shows that the use of the HSNet model proposed in this study for classification, with 95% confidence, results in significantly better classification prediction capability than the HSCNN model [8].

Table 13 presents a performance comparison of 15 research articles related to the classification of benign versus malignant pulmonary nodules sourced from the relevant literature for the years 2015–2023. These studies are listed according to seven indicators, including publication year, author, dataset, type of model, ACC, SEN, and AUC. This compilation provides a comprehensive overview of the state-of-the-art methodologies in the field. The data presented in bold in the table represent the best results. Due to variations in data quantities and verification methods in different studies, making meaningful comparisons becomes challenging. Nevertheless, the method proposed in this study continues to demonstrate outstanding performance.

**Figure 9:**
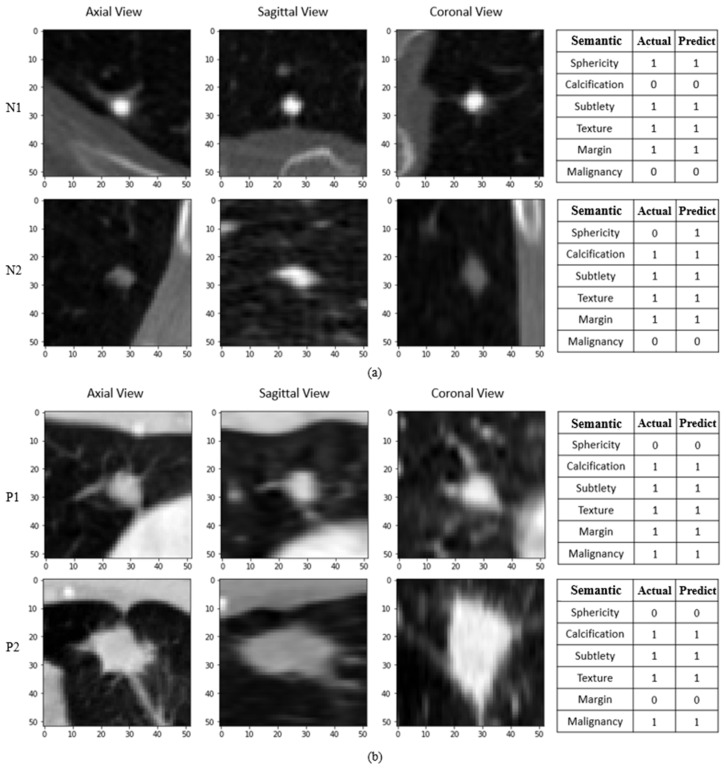
Demonstrating the nodule images and prediction results using HSNet model: (**a**) prediction results for benign nodule images; (**b**) prediction results for malignant nodule images.

This study uses the optimized method mentioned above to train model weights for image prediction. Figure 9 shows the results of the actual label and the predicted label in the images of the nodules using the proposed HSNet model. Figure 9a shows the predicted results of two benign nodules in the test set, labeled as N1 and N2, respectively. Figure 9b shows the predicted results of two malignant nodules labeled as P1 and P2, respectively. The three images on the left show the axial, sagittal, and coronal views of the nodule image, respectively. The axial view is a section constructed by the left, right, front, and back of the human body. The sagittal view divides the human body into left and right parts. The coronal view is a section divided into front and back parts along the longitudinal axis of the human body. By examining the sections, we can determine if the location of the nodule cut is correct and observe the symptoms from different angles to further prevent misdiagnosis when assisting doctors in diagnosis. The table on the right shows the original label and the predicted results of the images.

## 5. Discussion

The main purpose of this article is to develop a three-dimensional neural network model (HSNet) using interpretive three-dimensional lung CT images. This model can be used in a computer-aided diagnosis system to automatically identify lung cancer on CT images. The model is trained separately for five different semantic labels with distinct 3D feature extraction networks, with the aim of increasing the prediction accuracy of the model for each individual label. To optimize the HSNet model for six classification labels, several optimization methods were implemented, including cyclic learning rate, early stopping strategy, random weight averaging, and adjustment of model parameters. These methods were employed to refine the model’s performance and improve its ability to accurately classify lung cancer.

The results of this study indicate that the optimized HSNet model significantly improved the accuracy of malignant nodule testing from 93.22% to 97.84%, effectively improving the success rate of discriminating between benign and malignant nodules and improving the classification ability of the model for five different semantic labels. Compared to the results of Shen et al. [8], this paper demonstrated that using the proposed HSNet model to extract different features for each semantic label significantly increased the AUC values for each classification task. This confirms our hypothesis that training different semantic feature labels separately can increase the model’s prediction accuracy for each individual label in semantic-level classification tasks.

Regarding the automatic generation of medical diagnostic reports from CT images, the HSNet model, trained with semantic models using image features, was used for automatic diagnosis report generation. After evaluation using various assessment indicators, the similarity rate of the generated reports exceeded 90%. The results demonstrated that utilizing the proposed model’s features for training and generation improved the accuracy of subtitle evaluation by more than 20% compared to using the HSCNN model for generation. This validates the interpretation ability of the HSNet model after optimizing various semantic labels for the generation of reports.

This study has several limitations. The semantic labels of this study, as shown in Table 1, do not include certain imaging features that are highly relevant to malignant lung nodules, such as nodule size, shape, and location. Additionally, metadata such as sex, age, or family history are not included. The original semantic features had five or six levels, but to overcome data sparsity, they were converted into binary category labels, which can result in the loss of significant semantic information. Lastly, the malignancy label in the LIDC dataset is derived from manual radiological annotations and not pathological examinations. We also do not have data available to compare the nodule features over time. Thus, we can apply our results to the Lung Imaging Reporting and Data System (Lung-RADS), which is the most popular classification of lung cancer screening. These limitations can be improved by modeling large clinical annotated data sets. Therefore, the topic of model optimization remains to be investigated in the future.

## 6. Conclusions

The HSNet model proposed in this study utilizes the characteristics of nodules, including edge, sphericity, subtlety, texture, and calcification for semantic classification, and finally predicts the possibility of malignancy of nodules. By obtaining different features through various semantic labels, the classification ability of each label is improved. After adjusting various parameters and methods through different training strategies, such as cyclic learning rate and random weight averaging, the HSNet model achieves the best accuracy on the test set outside the training process. Furthermore, the semantic feature labels of the model are used to automate the generation of diagnostic reports. Computer-aided diagnosis is expected to assist doctors in the preliminary examination and the generation of diagnosis reports. In addition to the model’s classification prediction, a more interpretable explanation method is provided to assist in the judgment.

In future research, in addition to exploring different architectures of multimodal deep learning models, there is a need for a deeper understanding of the impact of various parameters and optimizations on the model. It is also anticipated that a larger amount of image data will be used to investigate the generalization ability of the model’s predictions.

## Figures and Tables

**Table 1:** Nodule semantic feature labels in the feature dataset.

Semantic Features	Original Level	Two Level
Malignancy	1. Highly unlikely	0. Benign
	2. Moderately unlikely	
	3. Indeterminate	
	4. Moderately suspicious	1. Malignant
	5. Highly suspicious	
Margin	1. Poorly defined	0. Poorly defined margin
	2.	
	3.	
	4.	1. Sharp margin
	5. Sharp	
Sphericity	1. Linear	0. Lesser roundness
	2.	
	3. Ovoid	
	4.	1. High degree of roundness
	5. Round	
Subtlety	1. Extremely subtle	0. Poor contrast between nodule and surroundings
	2. Moderately subtle	
	3. Fairly subtle	
	4. Moderately obvious	1. High contrast between nodule and surroundings
	5. Obvious	
Texture	1. Non-solid	0. Non-solid internal density
	2.	
	3. Part Solid	
	4.	1. Solid internal density
	5. Solid	
Calcification	1. Popcorn	0. Presence of calcification
	2. Laminated	
	3. Solid	
	4. Non-central	
	5. Central	
	6. Absent	1. Absence of calcification

**Table 2:** Comparison of parameter adjustment results of HSNet model.

No	Iteration	Optimizer	SWA	Test Accuracy
Run1	300	SGD	No	96.98%
Run2	300	Adam	No	97.08%
Run3	300	SGD-CLR	No	97.17%
Run4	300	SGD-CLR	Yes	97.65%
Run5	300	RMSProp-CLR	Yes	97.18%

**Table 3:** Test accuracy of four experiments using HSNet model.

No	Training Set	Validation Set	Test Set	Test Accuracy
Exp1	Fold2 + Fold3	Fold4	Fold1	97.84%
Exp2	Fold3 + Fold4	Fold1	Fold2	96.61%
Exp3	Fold4 + Fold1	Fold2	Fold3	96.52%
Exp4	Fold1 + Fold2	Fold3	Fold4	96.43%

**Table 4:** AUC of four experiments using HSNet model.

Semantic	Test1	Test2	Test3	Test4
Sphericity	0.926	0.942	0.944	0.905
Margin	0.954	0.966	0.967	0.965
Subtlety	0.948	0.963	0.956	0.956
Texture	0.982	0.978	0.994	0.976
Calcification	0.990	0.999	0.996	0.992
Malignancy	0.991	0.980	0.988	0.977

**Table 5:** Performance of semantic classification using HSNet model.

Semantic	Accuracy (SD)	Sensitivity (SD)	Specificity (SD)
Calcification	0.9873 (0.006)	0.9966 (0.002)	0.9151 (0.041)
Margin	0.9207 (0.009)	0.9584 (0.016)	0.8129 (0.021)
Subtlety	0.9026 (0.014)	0.9290 (0.016)	0.8584 (0.032)
Texture	0.9685 (0.006)	0.9933 (0.002)	0.7889 (0.049)
Sphericity	0.8652 (0.021)	0.8796 (0.038)	0.8638 (0.033)
Malignancy	0.9685 (0.006)	0.9267 (0.029)	0.9823 (0.004)

**Table 6:** Paired-sample *t*-test of sphericity.

Test Set	HSNet AUC	HSCNN AUC	AUC Difference	Paired *t*-Test
Fold1	0.926	0.577	0.349	*p* = 0.0004 mean difference 0.3207 95% CI [0.2635, 0.3780]
Fold2	0.942	0.590	0.352	
Fold3	0.944	0.640	0.304	
Fold4	0.905	0.627	0.278	

**Table 7:** Paired-sample *t*-test of margin.

Test Set	HSNet AUC	HSCNN AUC	AUC Difference	Paired *t*-Test
Fold1	0.954	0.829	0.125	*p* = 0.0037 mean difference 0.1725 95% CI [0.1059, 0.2391]
Fold2	0.966	0.739	0.227	
Fold3	0.967	0.800	0.167	
Fold4	0.965	0.794	0.171	

**Table 8:** Paired-sample *t*-test of subtlety.

Test Set	HSNet AUC	HSCNN AUC	AUC Difference	Paired *t*-Test
Fold1	0.948	0.823	0.125	*p* = 0.0002 mean difference 0.1293 95% CI [0.1110, 0.1475]
Fold2	0.963	0.817	0.146	
Fold3	0.956	0.830	0.126	
Fold4	0.956	0.836	0.120	

**Table 9:** Paired-sample *t*-test of texture.

Test Set	HSNet AUC	HSCNN AUC	AUC Difference	Paired *t*-Test
Fold1	0.982	0.847	0.135	*p* = 0.0005 mean difference 0.1642 95% CI [0.1325, 0.1960]
Fold2	0.978	0.801	0.177	
Fold3	0.994	0.826	0.168	
Fold4	0.976	0.799	0.177	

**Table 10:** Paired-sample *t*-test of calcification.

Test Set	HSNet AUC	HSCNN AUC	AUC Difference	Paired *t*-Test
Fold1	0.990	0.946	0.044	*p* = 0.0049 mean difference 0.0418 95% CI [0.0241, 0.0594]
Fold2	0.999	0.969	0.030	
Fold3	0.996	0.940	0.056	
Fold4	0.992	0.955	0.037	

**Table 11:** Paired-sample *t*-test of malignancy.

Test Set	HSNet AUC	HSCNN AUC	AUC Difference	Paired *t*-Test
Fold1	0.991	0.987	0.004	*p* = 0.0469 mean difference 0.0040 95% CI [0.0001, 0.0079]
Fold2	0.980	0.976	0.004	
Fold3	0.988	0.987	0.001	
Fold4	0.977	0.970	0.007	

**Table 12:** Significance test of performance between HSNet and HSCNN [8].

Semantic	HSCNN [8] AUC (SD)	HSNet AUC (SD)	Ps Value
Calcification	0.930 (0.034)	0.994 (0.004)	7.717
Margin	0.776 (0.033)	0.963 (0.005)	12.798
Subtlety	0.803 (0.015)	0.956 (0.005)	10.835
Texture	0.850 (0.042)	0.983 (0.007)	11.084
Sphericity	0.568 (0.015)	0.929 (0.016)	19.182
Malignancy	0.856 (0.026)	0.984 (0.006)	10.877

**Table 13:** Performance comparison of state-of-the-art methods for lung nodule classification.

Year	Author	Dataset	Model	ACC (%)	SEN (%)	AUC (%)
2023	Our Method	LIDC-IDRI	3D-White Box	**96.85**	92.67	98.40
2023	Zhang and Zhang [28]	LUNA16	3D-Black Box	92.75	-	-
2022	Halder et al. [29]	LIDC-IDRI	2D-Black Box	96.10	96.85	**99.36**
2022	Donga et al. [30]	LIDC-IDRI	2D-Black Box	95.67	91.00	-
2021	Zhang et al. [31]	LUNA16	3D-Black Box	92.40	87.00	-
2020	Agnes et al. [32]	LIDC- IDRI	2D-Black Box	-	81.00	94.40
2020	Liu et al. [33]	LIDC-IDRI	3D-Black Box	90.60	83.70	93.90
2020	Xia et al. [34]	LIDC-IDRI	3D-Black Box	91.90	91.30	-
2020	Ali et al. [35]	LIDC-IDRI	2D-Black Box	96.69	**98.10**	99.11
2019	Shen et al. [8]	LIDC- IDRI	3D-White Box	84.2	70.5	85.6
2019	Al-Shabi et al. [36]	LIDC-IDRI	2D-Black Box	88.46	88.66	95.62
2019	Al-Shabi et al. [37]	LIDC-IDRI	2D-Black Box	92.57	92.21	93.15
2018	Dey et al. [38]	LIDC-IDRI	3D-Black Box	90.40	-	95.48
2017	Nibali et al. [39]	LIDC-IDRI	2D-Black Box	89.90	91.07	94.59
2016	Shen et al. [40]	LIDC-IDRI	3D-Black Box	87.14	77.00	93.00
2015	Kumar et al. [13]	LIDC-IDRI	2D-Black Box	75.01	83.35	-

## Data Availability

The data utilized in this study are sourced from the publicly available dataset, LIDC-IDRI.
